# Deaf older adults’ experiences of support from a mobile old-age care team providing support in Swedish sign language

**DOI:** 10.1186/s12877-025-06675-1

**Published:** 2025-12-15

**Authors:** Elin Karlsson, Yashar Mahmud, Susanne Andersson, Linda Jonsson, Åsa Gustavsson, Sofia Kjellström, Sofi Fristedt

**Affiliations:** 1 Audiological Research Centre, Faculty of Medicine and Health, Örebro University, Örebro, Sweden; 2 School of Health Sciences, Faculty of Medicine and Health, Örebro University, Örebro, Sweden; 3 School of Communication Sciences and Disorders, Western University, London, ON Canada; 4 School of Health and Welfare, Jönköping University, Jönköping, Sweden; 5 Department of Leadership and Command & Control, Swedish Defence University, Stockholm, Sweden; 6 The Association of the Deaf and Sign Language Users in Jönköping County, Jönköping, Sweden; 7 Department of Health Sciences, Faculty of Medicine, Lund University, Lund, Sweden

**Keywords:** Deaf, Gerontology, Intervention, Older adults, Psychosocial support

## Abstract

**Introduction:**

To address communication barriers, minimise social isolation, prevent psychosocial illness and increase the independence of Deaf older adults, a mobile care team consisting of Deaf assistant nurses using sign language was initiated and developed by a nongovernmental organisation in a region in southern Sweden.

**Aim:**

To describe Deaf older adults’ experiences receiving support from an NGO-initiated mobile old-age care team for Deaf and sign language-speaking older adults in Sweden.

**Methods:**

A series of 15 individual interviews with four Deaf older adults were analysed via content analysis.

**Results:**

Support from the mobile care team was appreciated, as illustrated by the following categories: *support in everyday activities*, *communication supported and enabled* and *support for psychosocial well-being*. The care team facilitated communication using sign language. For example, they enabled in-depth communication and information sharing and supported older adults in expressing opinions and thoughts to authorities and regular care staff. Increased communication supported psychosocial well-being, independence, and feelings of safety.

**Conclusion:**

A sign language mobile care team that is well familiar with Deafness as a culture rather than a hearing disability is highly valued by Deaf older adults in need of home or residential care later in life. It also shows that access to a sign language mobile care team leads to increased psychological wellbeing and happiness among Deaf older adults, as well as to their increased participation in decision-making concerning various aspects of their lives.

**Supplementary Information:**

The online version contains supplementary material available at 10.1186/s12877-025-06675-1.

## Introduction

Ageing impacts communication in several ways not only for the general population but also for Deaf^1^ older adults. For Deaf older adults, a reduced ability to lip-read due to impaired vision is one negative impact of ageing, alongside motor function impairments affecting them when they use sign language. These impairments leading to changes in communication have led Deaf older adults to be marginalised due to a lack of a common language with hearing populations, increasing the risk of exclusion and isolation. These difficulties, in turn, lead to loneliness, depression, mental distress, and lower (physical and psychological) quality of life compared to hearing peers. [1–3]. When interactions with health or social care staff are restricted by a lack of a common language, Deaf older adults feel frustrated, may experience resignation and dependence [4]. Moreover, such restrictions may negatively impact health later in life [5].

Historically, Deaf people have been excluded from evaluation even for interventions made for this target group [6]. Several studies have been conducted in the field of dementia care, both from the perspective of Deaf older people with dementia and caregivers. These studies’ findings highlight the importance of communication to reduce social isolation (e.g.,[7, 8]). However, as communication is impacted by the cognitive changes seen in dementia [9, 10], the situation is not fully transferable to a population without cognitive impairment. Thus, there is a need to investigate the experiences of Deaf older people receiving care beyond dementia settings.

In Sweden, there are no statistics concerning the number of Deaf older adults, but in total, approximately 30,000 Deaf individuals use Swedish sign language [11]. In Sweden, approximately 17% of the population is over 65 years of age [12], and compared with the number of Deaf people, approximately 5,000 Deaf people are estimated to be 65 years and older. Deaf older adults are, as the general population of older adults, a heterogeneous group, and their needs and wishes concerning support from health and social care providers differ. The main language for Deaf older adults is Swedish Sign Language (hereafter termed sign language). Sign language is not the same as spoken Swedish, and sign language has different grammar, words, and syntax [11, 13]. As sign language is a language of its own, communication barriers occur in the same way as for other language minorities. It is also a risk that Deaf older adults misunderstand messages not given in their native language, that is, when they lip-read spoken language or when they read written language [13].

Beyond sign language, the Deaf community and the Deaf clubs are valued by Deaf people. Deaf clubs include group activities and meetings organised by Deaf people, such as events, sports, social engagement, and support. As society is rarely an inclusive space for Deaf people, Deaf culture and Deaf clubs are highly important, as they offer possibilities for communication in sign language, social engagement and the exchange of information [11, 14, 15]. In fact, community engagement and social support are determinants of healthy ageing across ageing populations [5]

Earlier research from other geographical contexts, such as the U.S. (e.g., [4]), revealed that Deaf older adults need more support than their hearing peers do, for example, with respect to activities in daily living (ADL). In Nordic as well as other European contexts, more recent research on old age care for Deaf older adults is lacking. Previous studies have shown that the need for support is based on life-span dependence, communication difficulties and noncommunicative issues [16]. This often means that these individuals need extra support and are at risk of isolation and a lower quality of life. Social participation is important, as individuals who regularly take part in activities (such as social activities and going to Deaf clubs) experience a higher quality of life than do Deaf peers who withdraw from these kinds of activities [2]. Moreover, Deaf older adults are at increased risk of loneliness compared with their hearing peers, with consequences such as decreased mental health and lower quality of life [1, 3].

To address these communication barriers, minimise social isolation, prevent psychosocial illness and increase the independence of Deaf older adults, a mobile care team consisting of Deaf assistant nurses using sign language was initiated and developed by a nongovernmental organisation (an NGO, i.e., a regional Deaf Club). The mobile home and care team (called the mobile care team) for Deaf and sign language-speaking older adults acted in a specific county of Sweden. The mobile care team was a pilot intervention implemented through a project and designed as a complement to regular support from old-age care provided to community-dwelling older adults or in residential homes. The intervention was implemented by the NGO and not by the researchers of the current study. The mobile care team provided support based on the individuals’ needs, and the Deaf older adults could decide themselves if and for what purpose they wanted this support. The aim of the mobile care team was to 1) provide support with the same interventions as the regular old-age care staff (IADL and, when needed, ADL) and 2) provide support with other activities not included in the support provided by the regular old-age care staff. For example, to have conversations, to provide and explain information, to help identify Deaf individual’s opinion and to explain it to non-signing staff, managers, or municipality officials. The mobile care team visited the Deaf older adults who opted to receive such support between one and three times a week.

As the mobile care team is the first of its kind and has neither been tested nor evaluated, it is important to evaluate the project from different perspectives. Investigating how older adults receive culturally valid support in their first language is also important. This trend urgently needs to be overcome to combat the marginalisation of this group. Therefore, the aim of the current study is to describe Deaf older adults’ experiences receiving support from an NGO-initiated mobile old age care team for Deaf and sign language-speaking older adults in Sweden.

## Materials and methods

### Design

The study utilised a qualitative design based on individual interviews with Deaf older adults. Ethical approval was obtained from the Swedish Ethical Review Authority (20200421, DNR: 2019–06372 and 20221017; DNR: 2022–05310-02). The study adhered to the Declaration of Helsinki, and, accordingly, all participants gave their written informed consent upon reading the information letter, which was also conveyed to them in sign language. Given the complex nature of Deafness and the challenges in the implementation of informed consent and data collection because of issues in communicating with participants [17], see also [18], we relied on Deaf and native sign language assistant nurses and research assistants to convey the content of the information letter to the participants.

### Participants

Six deaf older adults, all of whom were in contact with the mobile care team during autumn 2022, were invited to participate in the study and were informed in sign language about the study by staff from the mobile care team. The staff of the mobile care team delivered the information in written and signed formats. Additionally, the informed consent form was translated into sign language by both the staff from the mobile care team and the research assistants and was signed prior to the interviews. Voluntary participation was emphasised. Six agreed to participate from the beginning, but two did not want to take part in further interviews referring to health issues and were excluded from the study, resulting in 15 interviews with four participants (Table 1). All the participants were Deaf and used sign language as their first language. Two of the participants were women, two were men, and all lived in the same county in Sweden. All participants had support from old-age care, either home care or residential care, either by the municipal or private sector, and all participants had support from the mobile care team. Three participants were community-dwelling and had regular (daily or weekly) support from home care, and one participant lived in a residence home for older adults.

**Table 1:** Participant information

Participant ID	Gender	Age	Number of interviews
1	Female	73	4
2	Female	84	4
3	Male	88	4
4	Male	77	3

### Data collection

Prior to the data collection, a semi-structured interview guide, consisting of questions concerning support needed in everyday life activities and social life, was developed in Swedish written language. The guide was constructed for this study and has not been used previously. It was developed by the researchers and in collaboration with the research partners (coauthors and research assistants SA, LJ and coauthor and project leader coordination the mobile care team ÅG) The collaboration with the research partners increased not only the language understandability but also the relevance and trustworthiness of the study [19]. The guide also targeted how the older adults communicated in everyday life situations, specifically with non-sign language persons. The participants’ experiences of safety and security, as well as support from social care staff and social care situations, were also included. Finally, during the interviews, the Deaf research assistants asked the participants for further information and their suggested changes or improvements in any of the areas discussed during the interviews. The collaboration with the research partners enabled us to adapt the questions to work in sign language, and the interviewer asked complementary questions based on the participants’ responses to gain a deeper understanding. The interviews were conducted in sign language, in the participant’s private home or room in a care home, by a Deaf research assistant, and the interviews took between 8 and 54 min (mean 22 min). A process consent approach [20, 21] was used and before starting each interview, the research assistants (Deaf and using sign language) repeated the information about the study’s aims and obtained informed consent. All the information was also provided in written format for the participants. All the participants were invited to ask questions in sign language prior to and during the data collection, and the information was repeated when needed.

The interviews were conducted on four separate occasions for four of the participants (autumn 2022, spring 2023, autumn 2023 and spring 2024). Two Deaf research assistants were part of the data collection: the first conducted the first two series of interviews (i.e., autumn 2022 and spring 2023), whereas the second conducted the last two series of interviews (i.e., autumn 2023 and spring 2024); both were care professionals. All interviews were conducted in sign language by the Deaf research assistants, supported by the interview guide, in the participants’ homes. To address the challenges in conducting, recording, and transcribing interviews [22], the Deaf and native sign language research assistants had previous experience in research data collection and in video recordings.

### Data analysis

The video recordings were transcribed and translated verbatim from sign language into written Swedish by the respective research assistants. Following each interview occasion, one of the researchers and the respective research assistant jointly reflected on the transcripts to confirm that they were understandably and adequately translated into Swedish and to support trustworthiness. The data analysis started when three rounds of data were collected. The fourth round of interviews was completed during the same time period, and these transcripts were also added to the analysis.

Owing to the small sample of participants and the focus on understanding their responses [23, 24], the Swedish transcribed interviews were analysed via content analysis, according to Lindgren et al. [25]. The stepwise analysis was independently performed by two researchers (i.e., first, and last coauthors) and began with several readings of the interviews.

The analysis started with identification of meaningful units corresponding to the aims of the current study. Next, the meaningful units were condensed to meaning units, which were then coded. The coding was manifest and close to the text. From the codes, subcategories (e.g., support in activities other than those provided by regular home care/residential care; sign language makes it possible to access information; mobile care team interventions and sign language offer increased experience of safety) were interpreted and derived, and in the final step, the subcategories were abstracted into, as listed in the next section, 3 main categories. No joint overall theme was found or identified.

The analysis process started with two authors (E.K. and S.F.) analysing the data from the first round of interviews with the goal to try out coding and our data matrix, created in the software NVivo version 14. The results were then compared and discussed to ensure trustworthiness of the analysis and based on this, the data matrix and the initial coding were adjusted. In the next step, the following two rounds of interviews were analysed and, finally, the fourth round of interviews were added to the analysis. By adding one round of interviews at the time, it became clear that the number of new codes decreased for each round of interviews confirming saturation, also important for trustworthiness [19], it also strengthened the analysis and indicated sufficient broadness to support minimizing overlaps and the risk of having to many categories. When discrepancies in the analysis were identified between the two coauthors performing the data analysis, for example the number of codes or broadness of a category, the coding and interpretation were discussed between the authors, and the coding was then continued together to identify any similarities within as well as differences between categories. The final results represented the full transcripts well, strengthened the confirmability of the analysis – something that was also confirmed by the other members of the research group. In some cases, when transcripts were unclear or could be interpreted in more than one way, the researchers consulted the Deaf research assistants for clarification. This was also a strategy to retain quality and trustworthiness and minimise bias in the analysis. In addition, while the initial coding and analysis were conducted independently by two researchers (i.e., the first and last coauthors), all coauthors were actively involved in collaboratively shaping the presentation of the findings. When the analysis was complete, the contents of the subcategories and categories were described in English in parallel with the writing of the manuscript*.* The quotes displayed in the manuscript were translated from Swedish transcripts to English, mostly word by word, but when needed, they were adapted to make sense in the English language while remaining true of the original content. All analyses were conducted via the software NVivo version 14.

### Reflexivity and preunderstanding

When designing the study and analysing the data, the coauthors worked closely with the Deaf project leader, who coordinated with the mobile care team. To integrate knowledge about the Deaf culture and sign language into the project, it was coproduced and codesigned by the researchers, the Deaf coordinator of the home care team and the research assistants. This process gave the researchers (all hearing and not fluently in sign language) insight into the population and Deaf culture. The first coauthor has basic knowledge about Swedish sign language and Deaf culture and a research background in audiology, gerontology, and disability research, which might have biased the results. Similarly, the last coauthor, who is also part of the analysis, has deep knowledge of gerontology. The preliminary findings were discussed among all coauthors (including the Deaf mobile team coordinator and research assistants), and this discussion was used to support adequate reporting, interpretation, and trustworthiness.

## Results

The content analysis initially resulted in 84 codes, which were divided into 11 subcategories (see Table 2). The subcategories constituted three categories: *support in everyday activities*, *communication supported and enabled*, and *psychosocial wellbeing*.

**Table 2:** Overview of the categories and subcategories

Category	Subcategory
Support in everyday activities	• Support in other activities than the ones provided by the regular home care/residential care
	• Support in the same activities as the regular home care/residential care provides
	• The mobile care team preferred over regular home care/residential care
Communication supported and enabled	• The mobile care team and use of sign language enable communication
	• Sign language makes it possible to access information
	• The mobile care team provides support in communication with caregivers and authorities
	• Sign language makes it possible to make decisions
	• The mobile care team teaches regular home care/residential care staff sign language
Support for psychosocial wellbeing	• To meet staff using sign language increases happiness and joy of life
	• The mobile care team offers continuity and has time
	• The mobile care team interventions and sign language offer increased experience of safety

### Support in everyday activities

The mobile care team mostly provided the same support as did regular home care/residential care, but the mobile care team also offered support beyond that, and their efforts were very appreciated.

#### Support in activities other than regular home care/residential care

Both the regular old-age care staff and the mobile care team provided support in cleaning, grocery shopping, washing the dishes, and taking walks and taking out the garbage. Most participants were overall happy with the support from both instances, but all preferred to receive assistance from staff using sign language, as they could communicate when taking a walk or receiving support in the home. All the participants noted that the regular home care staff less often asked about what kind of support the participants wanted than did the residential home staff and the mobile care team did, and they explained that it was a combination of communication barriers and time. One participant expressed the following: “*They [the mobile care team] help me to clean, dust, and take the garbage out. I find it very nice of them to support me*” – Participant 2. The same participant did not trust the regular old-age care staff and did not want them to clean her home even though support was needed.

#### Support in the same activities as regular home care/residential care provides

When evaluating mobile care team support, the participants were happy to receive support in extended activities not regularly included in home care services, such as cleaning the closet, replanting flowers, helping with embroidery, and obtaining support to go out, for example, to go shopping or go for a trip in nature. The support was adapted based on the participants’ wishes and everyday activities and therefore included many different things. *“They support me with laundry and cleaning… [pointing at the flowers in the window] the mobile care team help me with them*” – Participant 4

#### The mobile care team preferred over regular home care/residential care

The participants living in their home, receiving care from the home care team, expressed that they preferred support from the mobile care team, and one participant now received more support from the mobile care team and less support from the regular old-age care.

“*I don’t receive much support from the hearing staff anymore [regular home care team]. I just receive support with cleaning every third week and grocery shopping every second week, nothing more. I receive most support from the mobile care team now, three times a* week.” – Participant 1

### Communication supported and enabled

A key strength of the mobile care team is the support enabled by communication in sign language. This increased access to information, enabled decisions about their own situation and, overall, facilitated communication. The fact that the mobile care team also taught regular home care/residential care staff sign language was also appreciated.

#### The mobile care team and the use of sign language enable communication

When interacting with regular old-age care staff, communication quality is highly dependent on the relationship with and competence of a specific staff member, according to some participants. They all expressed some level of barriers in communication, and as the staff did not know any sign language, most communication was spoken/lip-reading or consisted of writing on a paper. The communication was therefore experienced as limited, and there was no room for small talk or questions to express health conditions or one’s preferences concerning support.

*“He [a regular home care staff member] talks in an unclear way, and it is hard to lip-read. Then, I feel helpless. The other staff member, a woman, talks in a clear way and then I understand. Even though they don’t know sign language, the communication works* well.” – Participant 3

Notably, after receiving support from the mobile care team, some of the participants started to change their views on communication. Before the arrival of the mobile care team, all the participants agreed that spoken/lip-read communication with hearing staff was the only opportunity. The possibility of communicating with a Deaf staff member via sign language was described as giving increased happiness and that it was much appreciated to be able to have a conversation with someone. “*They [the mobile care team] sit and talk to me, and we go out for walks. If the weather is bad, we sit here and talk for approximately 30 min, then they leave.*” – Participant 3

“*I must say that I am very happy about the mobile care team. It is easier to express oneself in sign language [hand on her heart] … When communicating with the others [regular home care staff], there are barriers, and I feel that it is impossible to have an in-depth conversation like I can with the mobile care team.*” – Participant 1

#### Sign language makes it possible to access information

Most of the participants did not communicate with anyone during the day, even if some had a few friends visiting and others made video calls to friends. Most of the participants expressed that they missed going to Deaf clubs and taking part in the Deaf culture, as it was important for their social life. They described that meeting other Deaf individuals allowed them to receive news, ask questions and socialise. However, the participants described that the mobile care team was a substitute for the Deaf club, as the mobile care team staff could give them updates from the Deaf community. Participant 1 said, “*When they have been at the Deaf club, they tell me all the news. They tell me, and I can know what’s going* on.”

All the participants described being dependent on others (e.g., their children, relatives) to pass forward information, complaints, and questions to regular old-age care staff, managers, and authorities. The same was described concerning receiving information, as the participants described that they could neither access information from the regular old-age care staff nor fully understand written information from, for example, the municipality. All but one participant (one participant still preferred support from a significant other) experienced a positive change when they started to receive support from the mobile care team, as they could obtain information directly in sign language and had the possibility to ask questions. They reported that when they could take part in information, it gave them an opportunity to make their own decisions concerning their lives. It also made them aware of their right to receive support and take part in home adaptations available for all older adults in society. Most of the participants expressed that it was very supportive to have someone who could explain things and answer questions. This included both explanations of written information and clarifications of other information and news.

“*I asked the mobile care team staff a question, and then we discussed it together. Next, the mobile care team contacted the authorities and asked for support, and they helped. I got a response, and everything was clear and approved. They [the mobile care team] help me a lot.*” – Participant 3

“*They [the mobile care team] are good, and I am happy. They sit there and tell me different things. We handle different matters and then they talk with the regular staff members about how I want things to be done. We discuss a lot.*” – Participant 3

#### The mobile care team provides support in communication with caregivers and authorities

All the participants reported that without information and the ability to express their own thoughts, wishes and opinions, their independence was limited. With respect to larger life decisions, relatives could be involved, but this was more difficult concerning decisions in everyday life. In everyday life situations and meetings with regular home care/residential staff, owing to communication barriers, it was difficult to express wishes, leading to less initiative and independence. The participants could see a clear change after starting to receive support from the mobile care team, as they enabled the information needed to make their own decisions, in turn making them feel more independent. A clear example of this was when the mobile care team staff made video calls when grocery shopping. This decreased the risk of misunderstanding; mistakes were limited, and the participants felt involved in the decision-making process.

“*Yes, I prefer the mobile care team rather than the hearing regular staff. For example, when they [the mobile care team] go grocery shopping, they make video calls and ask what groceries I prefer. They show me what I can choose from, and I can see* directly.” – Participant 1

#### The mobile care team teaches regular home care/residential care staff sign language

Finally, all the participants were happy that the mobile care team staff taught the regular care staff sign language. This was experienced as helpful, as even though it did not lead to any deeper conversations, it was at least easier to use some signs to support communication with the regular old-age care staff. However, it was also clear that some staff did not want to learn, and due to staff turnover, it was difficult to implement sign language. “*One of the members of the mobile care team try to teach the regular home care staff sign language. One of the home care staff members has learned some sign language easily, and I like that* person.” – Participant 1

### Supporting psychosocial well-being

The mobile care team supported increased happiness and joy of life, a continuity in relationships and an experience of safety. The use of sign language improved quality of life.

#### Meeting staff using sign language increases happiness and joy in life

All the participants expressed feelings of happiness and joyfulness, were less depressed, and said they were laughing more thanks to the mobile care team meetings and access to communication.

“*I feel happy now; I am very happy for the mobile care team. It’s nice, and I feel more energised. Before [the mobile care team],] I felt sad and disappointed, but now I feel energised and happy. I always smile and feel happy when the mobile care team comes here.*” – Participant 1

#### The mobile care team offers continuity and has time

Another aspect mentioned by all the participants, especially when living in a residential home, concerned the lack of staff continuity. Owing to visits from several different regular staff members as well as staff turnover, communication and relation building became more difficult, in turn limiting trust. The communication often worked well with some people but less well with others, and two of the participants sometimes asked the regular care staff to leave, as the communication barriers were too extensive. The continuity given by the two mobile care team staff members was clearly appreciated. Moreover, they had more time than did the regular staff, which also supported relationships and communication.

*“Regular home care offers less, and it is hard; they are always in a rush and have many others to think about. The mobile care team staff take it easy, so there is a difference between them and the regular home care team.*” – Participant 1

#### The mobile care team interventions and sign language offer increased experience of safety

All the participants felt safe in their current home, and safety alarms contributed to their sense of safety. However, they described feeing even more safely after the introduction of the mobile care team. This was explained in greater detail by some of the participants and was based on several parameters related to communication: trust, independence and access to language and information. This experience concerned both regular staff using limited sign language and the mobile care team with full sign language. Two examples included receiving support related to medications and support in communication between nurses or other health care professionals. This support was expressed as making the participants feel safe and decreasing the risk of misunderstandings. Another example described by a participant was that she did not want to leave her room or eat in the dining area (placed in the same building) alone or with hearing peers, as it felt embarrassing and stigmatised. However, at the company of the mobile care team, the participants felt safe and had confidence in their ability to eat in the dining area. When asked about the future, all the participants expressed that signing home care/residential care staff was a priority to be able to communicate: “*I want a residential care home with access to staff using sign language. That would make me feel* safe.” – Participant 3

## Discussion

Overall, the results of the current study suggest that the participants, Deaf older adults, experienced positive support from the mobile care team, as illustrated by three categories: *support in everyday activities, communication supported and enabled* and *support for psychosocial wellbeing.* The Deaf older adults reported that they were able to communicate, receive information and express their thoughts in ways that were not possible before receiving support from the mobile care team. The mobile care team and the use of sign language gave the participants increased independence and a feeling of safety.

Illustrated more deeply in one of the categories, the possibility of communicating in sign language while receiving care was a key advantage of the mobile care team, according to the participants. Communication is a fundamental right [26], but the responses from the participants in the present study illustrate how this right is not honoured for Deaf older adults. The participants’ ability to communicate was clearly restricted, and the mobile care team staff was the only staff member with whom the participants could communicate. The in-depth communication about everyday things enabled by the mobile care team supported the participants shared decision-making, enhanced their well-being, and made them feel happier and less depressed. Previous studies have shown similar benefits of sign language communication for Deaf older adults with dementia [7, 9, 27, 28]. This is an important finding to consider in practice, as older adults with hearing loss are at risk of depression and isolation [29], and the risk is even greater for Deaf older adults [1, 3].

All the participants experienced increased psychosocial well-being and happiness and less need to sleep during the days when they had the mobile care team to communicate with them and when they looked forward to the meetings. To say a few words/lip-reading or write a note on a paper is not enough to receive social and communicative stimulation or to deliver person-centred and secure care. The use of written information clearly increases the risk of misunderstandings, as it is not the Deaf individuals’ first language [13, 30]. The social factors and activities are, despite their documented advantages [31], less often prioritised within the old age care needs assessment. Moreover, the home care and residential care staff prioritise support in more basic, personal activities. This can partly be explained by limitations in time and resources, whereas the Swedish Social Services Act [32] clearly supports participation and social activities.

As noted above, the mobile care team communication and approach made shared decision-making possible. For larger life decisions, such as where to live or economic issues, relatives were in charge or supportive. In regular care situations, due to a lack of a common language, home care/residential care staff made most of the everyday life decisions, as the staff could neither ask the participants of their view nor the Deaf older adults displayed their wishes. Despite these limitations, the older adults did not complain. Potentially as they did not have anything else to compare with. However, they clearly expressed being more involved and informed by the mobile care team service. There is a risk that Deaf older adults enter a more vulnerable situation again when the project funding for the mobile care team ends. Our findings also show that the service for Deaf older adults fails to fully satisfy Swedish laws and policies calling for equal health [33] and social care [32], and similar to what has been concluded for the general population of Swedish older adults, more comprehensive, codesigned, integrated and person-centered interventions are needed [34]. Person-centred care and health ethics include the right to self-determination. This means that every individual has the right to make decisions concerning their life, including receiving the information necessary to make informed decisions [35].

Similarly, the Deaf older adults had not previously noticed any information about their rights concerning societal support with transport or housing adaptations (e.g., railings, doorsteps). In line with the Swedish Social Services Act [32], the participants finally received this information in sign language from the mobile care team, and when the adaptations were made, they felt safer and less afraid of falling. Such adaptations are known to enable older adults to stay in place as well as to promote safety, independence, and quality of life for older adults in general [36, 37]. Moreover, it is a human right to be able to express opinions about things that affect one's everyday life and that these opinions should be respected [38]. In fact, citizens in Sweden have the right to an interpreter in such everyday life situations to support access to information and to express one’s opinion. The Swedish National Board of Health and Welfare report [38] is old, and although further investigations have been conducted by the government since 2008, no major change or improvement has taken place to support the communication of deaf older adults.

The support provided by the mobile care team consisted of the same activities as did the regular home care/residential care staff, but the support provided by the mobile care team also went beyond that. All participants in the current study missed meetings at the Deaf club, and the interventions by the mobile care team were experienced as partly a substitute and a chance to receive news and information. Access to the mobile care team also gave them an opportunity to talk to someone, which gave them happiness and the ability to share their thoughts, which was strongly restricted for all participants. Moreover, when interpreting the results, it is important to remember that it is not possible to compare mobile care team interventions with regular home care/residential care. Not all the activities in which mobile care team care is provided are available through ordinary old-age care. However, the mobile care team was never planned to be a substitute for regular care but rather a complement that included social aspects. However, in the end, some participants received most of the support from the mobile care team and less from the regular care team, whereas others, for example, those living in a residential care home, received most support from regular care staff and the mobile care team as a social complement. This variation might have impacted the results, as the context, type and amount of support differ between the participants.

### Implications for future practice

Our findings indicate that care and welfare organisations need to critically reflect on their activities and approaches linked to Deaf older adults. Disregard for Deafness and Deaf culture is common in the hearing community [39, 40], and it is important that care staff develop their knowledge and understanding of Deaf older people’s needs and circumstances. Care organisations also need to acknowledge the need to work beyond ‘doing business as usual’ and recognise the specific needs of Deaf older adults to support their right to equal care [41]. Building relationships and trust [42] as well as coproducing care with stakeholders [43] are important to promote health equity, not least in minority groups such as Deaf older adults [44]. The mobile care team in the present study was appreciated by Deaf older adults and could serve as a model to support health equity for this group. Since the group of Deaf older adults is small, it may, for example, be beneficial for several care organisations to join forces and engage a team based on the mobile care team model described in this article. It would also be beneficial for Deaf older adults to receive support from as few professionals as possible and have an individual contact person/assistant nurse who truly gets to know the older Deaf person and their needs and conveys this to their colleagues.

### Strengths and limitations

There are some methodological issues to consider, starting with the small sample of participants (*n* = 4). In qualitative research, the sample size is often smaller, as the focus is to collect rich and in-depth data and not statistical power [45]. In the present study, all Deaf older adults within the county who received support from the mobile care team were informed about the study and invited. Initiatives targeting older Deaf populations are rare and important to follow through with research, especially as Deaf individuals have historically been and are excluded from research [6]. Given the potential participants’ previous experiences of exclusion, we strived to create a safe environment, and the participants were invited after getting to know the mobile care team. Moreover, they obtained information about the study in sign language and had the possibility to ask questions, which is important, as written information is not enough, as highlighted in the present study results.

We conducted a series of interviews instead of one focus group to compensate for the low number of participants, as it was considered likely that the participants would be safer in sharing their thoughts when they were more acquainted with the interviewer and during the data collection process. Moreover, the participants were geographically spread, making a focus group design less feasible. This made it possible to ask a few questions at the time and to let the participants get to know the interviewer. In the end, almost no new information was added in the final round of interviews, indicating that most topics are covered. The analysis included longitudinal data, and as a result, it was possible to identify changes in experience with the mobile care team over time. This was a strength of the current study, as it made it possible to capture both experiences of when the mobile care team was new for the participants and when it was a well-integrated part of their everyday life.

Another strategy of the data collection process was to have Deaf signing interviewers avoid interpreters. Previous studies have shown that Deaf older adults, based on historical stigmatisation, are not always comfortable signing with hearing people or using their voice with unknown people [46]. Another factor for using Deaf and native language research assistants was their familiarity with Deaf culture, making it easier to ask relevant follow-up questions. Two interviewers were involved in the current data collection, one for the first round of interviews and the other for the remaining three rounds. The change in the interviewer had a positive impact, as the second interview asked more follow-up questions. On the other hand, the first round of interviews was a test round where the interview guide was tested, resulting in some adaptations. Relying on Deaf research assistants to conduct interviews was important in terms of cultural matching, which is important when translating materials, for example, questionnaires in general [47] but also specific when translating them into and from sign language [48]. We acknowledge that there might have been certain translation dilemmas [48], and one way to handle these dilemmas was to work closely with Deaf research assistants, who could validate signs when needed, as a sign might have several meanings and explain the interpretation in the transcribed material.

More research in this field is needed, and a longitudinal study with a larger sample size would be beneficial. However, it is important to remember that, as the population is limited, identifying larger groups of participants is challenging. The data analysis method of the current study was found to be suitable for this initial study investigating the experiences of receiving care from Deaf assistant nurses via sign language. However, in future studies, it would be interesting to include other perspectives and data analysis methods. We have added a sentence about this in the limitations section. It would also be important to investigate support from the mobile home care team from complementary perspectives, such as the perspective of staff and relatives, as well as to evaluate the cost-effectiveness of similar interventions.

## Conclusion

The present study targets a population rarely considered in research and shows that a sign language mobile care team that is well familiar with Deafness as a culture rather than a hearing disability is highly valued by Deaf older adults in need of home or residential care. It also shows that access to a sign language mobile care team leads to increased psychological wellbeing and happiness of participants, as well as increased participation in decision-making concerning various aspects of their lives. As a result, the participants expressed their desire for continued support from signing staff in the future. This is the first study to investigate how Deaf older adults experience support from a signing care team, and more research about Deaf older adults and their experiences with home care, residential care and communicative and social support is needed.

## Supplementary Information

Supplementary Material 1.

## Data Availability

Deidentified data are available upon request, in line with ethical approval.

## Footnotes

1 Deaf with capital D refers not only to the audiological status of the person but also to the culture and community of Deaf people often, but not always, using sign language as primary language.
