# Supplementary material for: Deaf older adults’ experiences of support from a mobile old-age care team providing support in Swedish sign language
Source: BMC Geriatr. 2025 Dec 15;26:79. doi: 10.1186/s12877-025-06675-1 (PMC12829045; doi:10.1186/s12877-025-06675-1)
Supplement: Supplementary file 1 — Supplementary Material 1. [file 12877_2025_6675_MOESM1_ESM.docx]

## Interview guide

Questions asked in order as relevant based on the ongoing dialogue.

General follow-up question:

- Please, provide an example
- How do you mean?
- Please, develop your thoughts

1. What help do you need with your everyday activities?
   1. When do you need help?
   2. How do you experience that help?
   3. What makes it easier when you need help?
   4. What makes it difficult when you need help?
2. What do you do when you need help, but there is no one around who knows sign language?
3. How do you experience the help you receive from regular old age care staff?
   1. What can they help you with?
   2. What can't they help you with?
   3. What do you want to be different?
   4. Do they ask you how you want to get help? In what way?
   5. How would you like help?
4. How do you experience the Mobile care team efforts?
   1. What can they help you with?
   2. What can't they help you with?
   3. Do you think they could help you with something else?
   4. Do they ask you how you want to get help? In what way?
   5. How would you like help?
5. What are the differences between the efforts of the hearing staff and the mobile care team?
6. In which activities would you like to participate in more?
7. Can you describe specific situations when people have not understood you?
   1. Who are the people who understand you?
8. Who decides with what and how you should get help?
   1. Have you been involved in deciding over your own care or support? In what way? If not, what do you want? How would you like support?
9. How do you like it here (where you are staying)?
10. How do you socialize with others?
11. Do you feel lonely? If so, when? How?
12. Do you feel included or excluded? If so, when? How?
13. Do you feel exposed? If so, when? How?
14. How safe are you here? What can make you feel safer?
15. Do you find it easy or difficult to understand written Swedish?
    1. What do you do when you receive information from authorities? Do you need help from someone and how does it work?
16. Is there anything you want to do (or be) differently to improve your situation?
    1. If so, what?
